# Successful Mitral Valve Repair for Single Leaflet Device Attachment Following Transcatheter Edge‐to‐Edge Repair: A Case Report

**DOI:** 10.1155/cric/6112171

**Published:** 2026-06-22

**Authors:** Takuro Makiura, Masahiro Daimon, Takahiro Katsumata

**Affiliations:** ^1^ Department of Thoracic and Cardiovascular Surgery, Osaka Medical and Pharmaceutical University, Takatsuki, Osaka, Japan, ompu.ac.jp

**Keywords:** case report, MR, MVP, SLDA, TEER

## Abstract

An 87‐year‐old man underwent transcatheter edge‐to‐edge repair (TEER), one MitraClip NT︎ from anterior commissure (AC), 8 months prior for severe mitral regurgitation (MR) due to functional etiology and A1 chordal rupture, complicated by heart failure. Following improvement in heart failure, he was discharged home independently 2 weeks postprocedure. However, 4 months ago, he experienced worsening heart failure, and echocardiography revealed recurrent severe MR due to single leaflet device attachment (SLDA) at P1, prompting a surgical intervention. Intraoperatively, after establishing cardiopulmonary bypass and inducing cardiac arrest, transseptal access revealed the MitraClip NT︎ firmly adhered to the P1 leaflet and extensive rupture of the A1 chordae. The MitraClip NT︎ and involved leaflet tissue were excised en bloc, the defect was repaired, artificial chordae were implanted, edge‐to‐edge repair of the AC region was performed, and a prosthetic annuloplasty ring was sutured in place, successfully eliminating regurgitation. Since MitraClip NT︎ becomes encapsulated by fibrous tissue within 2–3 months postimplantation, isolated removal becomes technically difficult in the late phase. Therefore, en bloc resection with the leaflet is necessary in cases of SLDA‐related recurrent MR. SLDA involving the posterior mitral leaflet is considered more amenable to mitral valve repair than that involving the anterior mitral leaflet. Patients selected for TEER generally have limited surgical tolerance; thus, minimizing operative and cardiopulmonary bypass times is essential. Surgical approach should be tailored based on leaflet morphology and clip location.

## 1. Introduction

Transcatheter edge‐to‐edge repair (TEER) is a transcatheter technique used to repair mitral valve regurgitation in high‐risk patients, such as elderly individuals, who have limited surgical tolerance. Although this procedure is less invasive than open‐heart surgery, certain patients eventually require surgical intervention due to mitral regurgitation (MR) recurrence or difficulty in maintaining MR suppression. Such treatment failure can be caused by single leaflet device attachment (SLDA). Since it is generally difficult to remove only the MitraClip from the mitral valve, mitral valve replacement (MVR) is often selected when SLDA occurs. Here, we report a case in which the SLDA was excised along with part of the mitral leaflet, and successful mitral valve repair (MVP) was achieved, leading to suppression of severe MR.

## 2. Case

**Figure 1:**
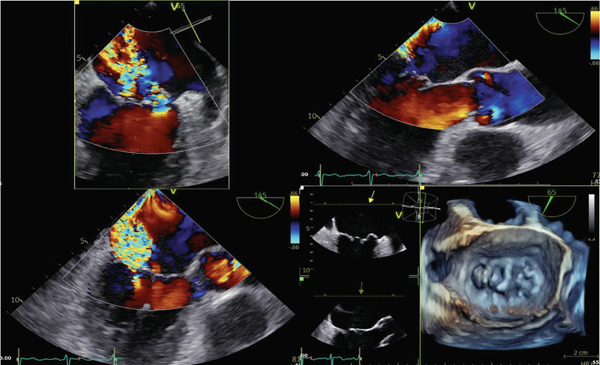
Pre‐TEER TEE. A dominant jet was observed between A1 and P1, extending along the PML to the base of the left atrium. A lateral jet was also seen in the commissural view. The jet persisted throughout systole without attenuation and was broad. Straight jets were also noted from A2‐P2 and A3‐P3, similarly showing no attenuation during systole. The length of PML measured 9 mm, and the length of AML measured 18 mm; the AML leaflet was mildly thickened and showed signs of degeneration (3.7 mm). The subvalvular apparatus appeared to be normal. The annular dimensions were: 35 mm at 0°, 38 mm at 60°, 21 mm at 135°; transverse × vertical diameter: 23 × 38 mm (3D); annular circumference: 90 mm; annular area: 7.5 cm^2^; mitral valve orifice area: 6.6 cm^2^; left atrial diameter: 51.6 mm. The PISA‐derived effective regurgitant orifice was 1.0 cm^2^, and the regurgitant volume was 135 mL. Pulmonary vein flow showed systolic reversal. The vena contracta was 1.0 cm.

**Figure 2:**
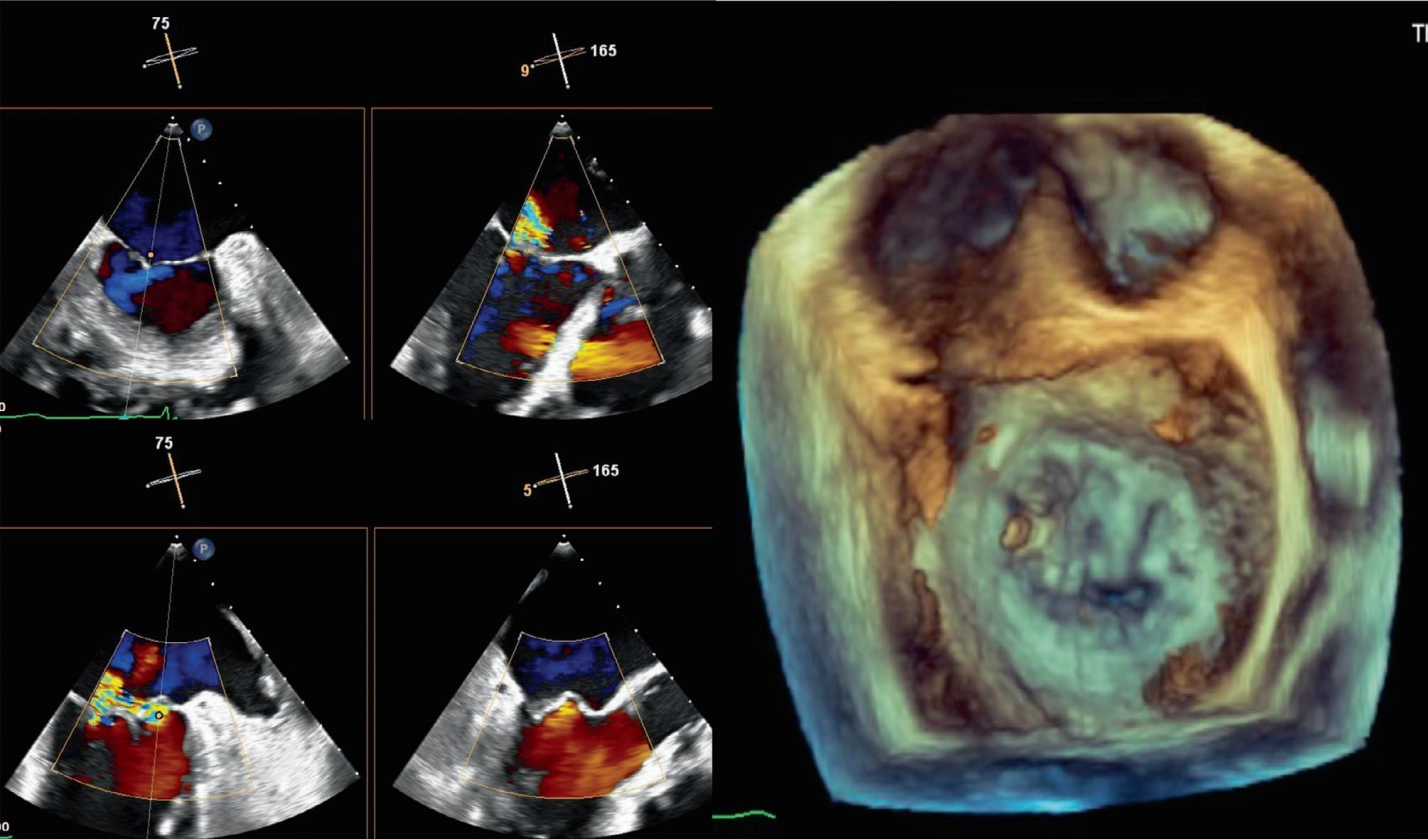
Post‐TEER TEE. A single MitraClip NT︎ was placed at the AC/P1 segment. MR on the AC side was adequately suppressed; however, residual MR remained on the A2/P2 side. Moderate MR was observed at the end of the procedure.

An 87‐year‐old man with a medical history of hypertension, diabetes mellitus, dyslipidemia, membranous nephropathy, prostate cancer surgery, bladder adenocarcinoma surgery, and recurrent duodenal ulcers was admitted to our institution with exertional dyspnea and diagnosed with congestive heart failure caused by moderate MR. After 18 days of medical therapy, his symptoms improved and he was discharged; however, he was readmitted 6 days later because of recurrent dyspnea. Because his valvular heart failure was refractory to medical treatment, he was considered a candidate for either open‐heart surgery or TEER. Given his advanced age and multiple comorbidities, TEER was selected as the initial treatment strategy. Preoperative transthoracic echocardiography (TTE) revealed chordal rupture with prolapse of A1 and AC segments and annular dilatation as the primary causes of MR. Clipping of the AC/P1 segment, where regurgitation was the most severe, was planned (Figure 1). Intraoperatively, the clip delivery system was inadvertently introduced into the left atrium through a patent foramen ovale. As a result, the clip delivery system assumed an aorta‐hugging trajectory, making grasping at the AC region difficult. When attempting to withdraw the MitraClip NT back into the left atrium, the device became entangled with the chordae, requiring in situ repositioning. The posterior leaflet was grasped first, followed by the anterior leaflet, and clipping was successfully completed. Although mild residual regurgitation remained at the AC region, overall MR was markedly improved (Figure 2). The postoperative course was favorable, and the patient was approved for ambulatory discharge 2 weeks after the procedure.

**Figure 3:**
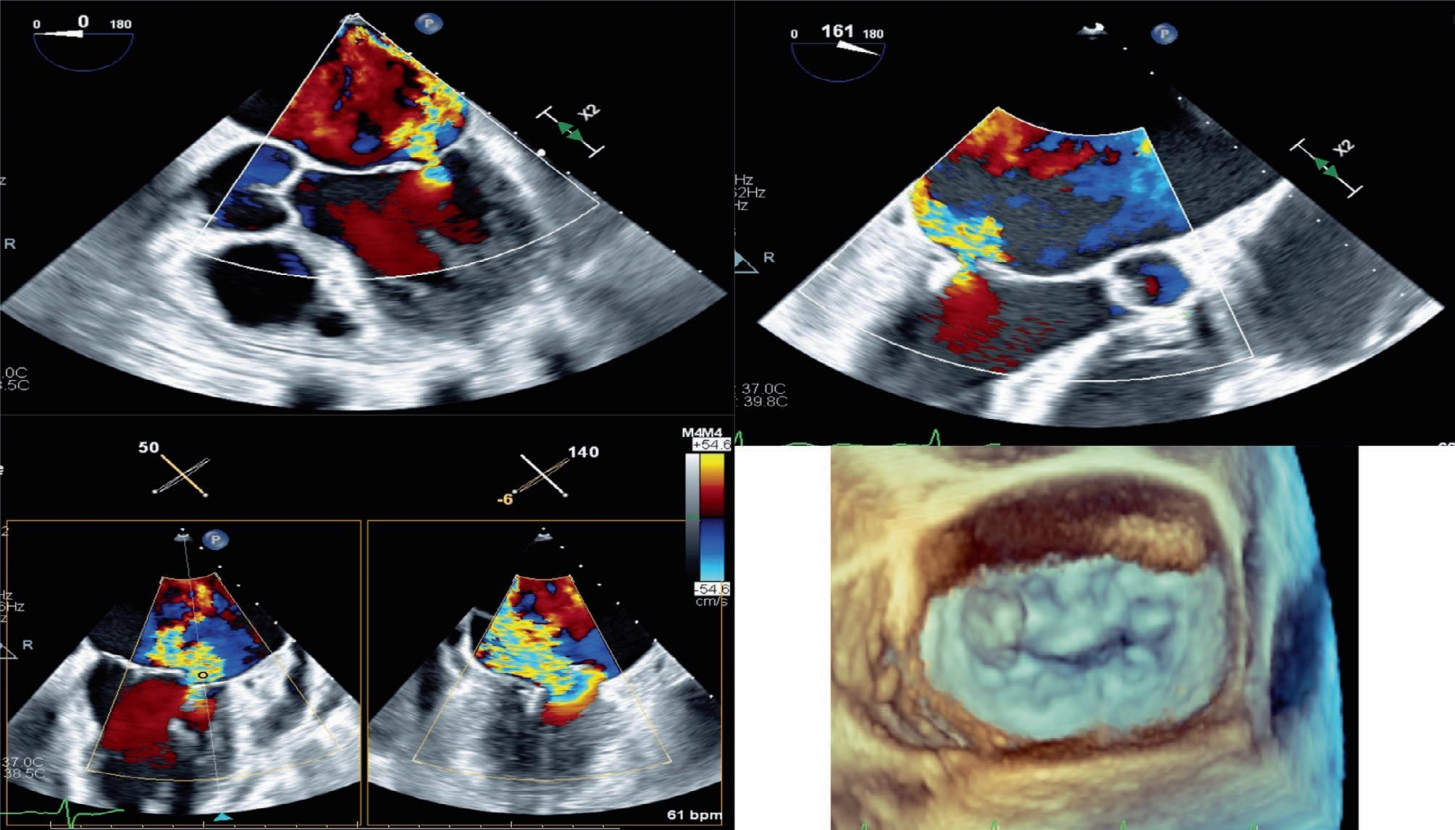
A broad jet with significant suction flow from the AC/A1 region reached the base of the left atrium. A straight jet with broad suction flow was also observed from the A2/P2 region. Detachment of the clip at the AC region was noted, along with prolapse of both the AC and A1 segments. Functional MR was present from A2/P2. Both the AML and PML showed evidence of tethering. TEER = transcatheter edge‐to‐edge repair, TEE = transesophageal echocardiography, AML = anterior mitral leaflet, PML = posterior mitral leaflet, and MR = mitral valve regurgitation.

However, 4 months later, heart failure recurred. TTE revealed recurrent severe MR due to SLDA, and the patient was referred to our department for surgical treatment (Figure 3). His medications included enalapril, empagliflozin, furosemide, and spironolactone for chronic heart failure; potassium‐binding resin and prednisolone for membranous nephropathy; and esomeprazole for recurrent duodenal ulcers. Laboratory findings showed renal dysfunction, mild anemia, thrombocytopenia, and elevated BNP. Pulmonary function testing showed restrictive impairment. Chest‐abdomen‐pelvis CT scans demonstrated ground‐glass opacities in the lungs that were consistent with heart failure. The Clinical Frailty Scale score improved from 7 before the MitraClip procedure to 5 at the time of admission for SLDA. The Society of Thoracic Surgeons score estimated a perioperative mortality risk of 10.5%. During the preoperative discussion of treatment strategies, we explained that repeat TEER would be less invasive but would primarily address functional MR; therefore, residual regurgitation from the prolapsing leaflet might persist, and there would also be a risk of recurrent SLDA, making the procedure potentially palliative. In contrast, surgical treatment with MVP or MVR could provide a more definitive solution; however, advanced age, renal dysfunction, and immunosuppressive therapy represented significant risk factors, with substantially higher risks of mortality and irreversible complications compared with standard surgical candidates. After careful consideration, the patient elected to undergo surgical treatment, acknowledging the high procedural risk in order to reduce the likelihood of recurrent heart failure.

**Figure 4:**
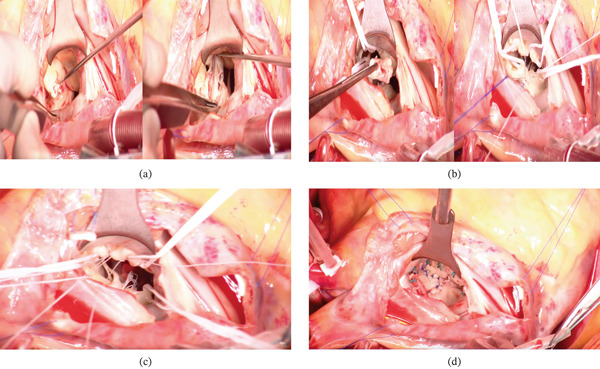
Intraoperative findings. (a) The MitraClip NT had adhered as a single mass to the P1 leaflet, and extensive chordal rupture was observed in A1. (b) The MitraClip NT was excised along with the leaflet tissue, the defect was closed, and three artificial chordae were implanted at the site from the posterior head of the anterior papillary muscle. (c) Two artificial chordae were placed to A1 and one to A2 from the anterior head of the anterior papillary muscle. (d) Edge‐to‐edge repair was performed at the AC region, and a 29‐mm Tailor Ring was sewn to the mitral annulus.

**Figure 5:**
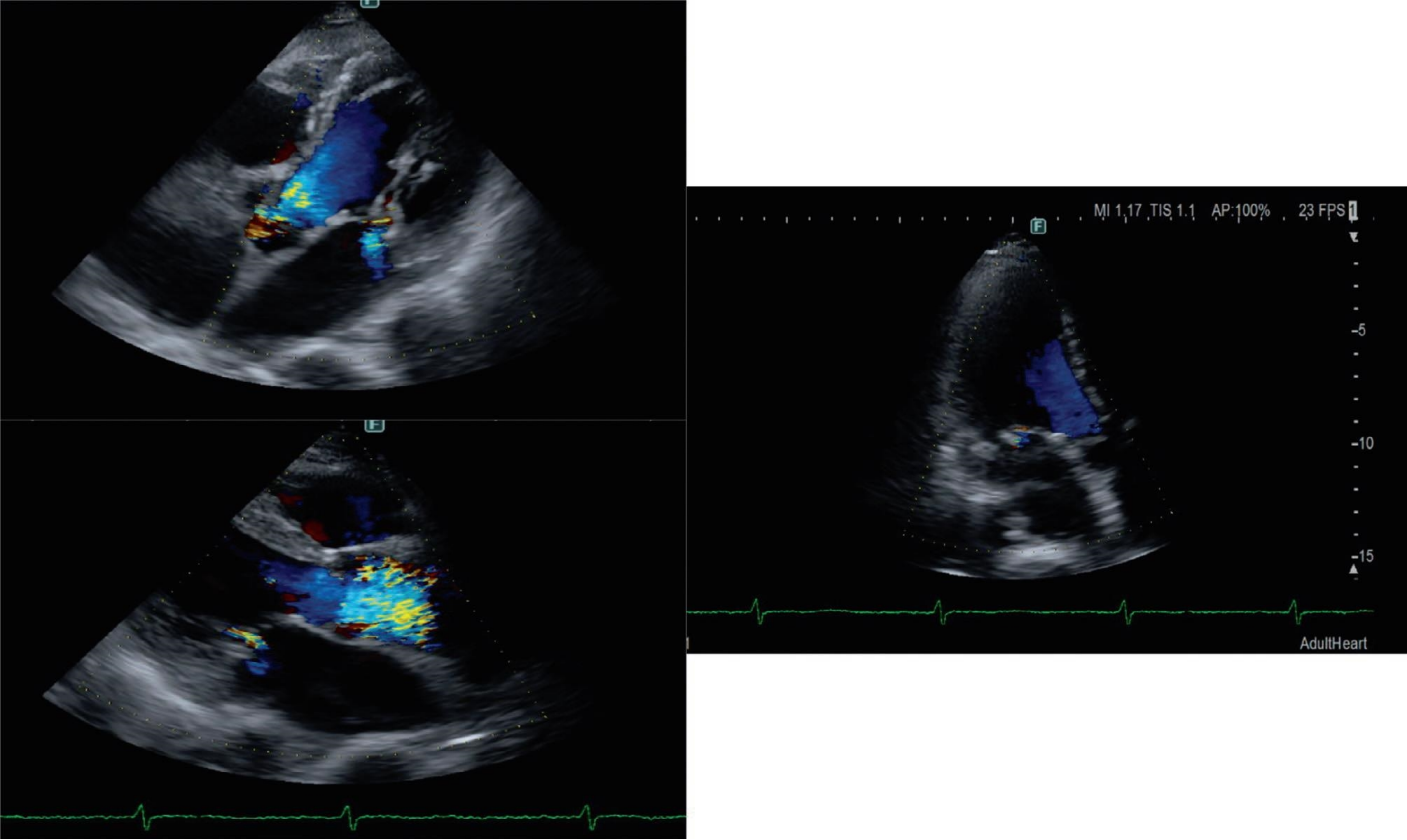
TTE after MVP MR was reduced to trivial levels.

During surgery, after establishing cardiopulmonary bypass and cardiac arrest, the mitral valve was examined via a transseptal approach. The MitraClip NT︎ had firmly adhered to the P1 leaflet and integrated with it. Extensive chordal rupture was noted at A1. The MitraClip NT︎ was excised en bloc with the posterior mitral leaflet (PML), and the defect was directly closed. Three artificial chordae were implanted from the posterior head of the anterolateral papillary muscle to P1. Two artificial chordae were placed from the anterior head of the same papillary muscle to A1, and one was placed from the anterior head to A2. AC was treated with commissuroplasty, and a 29‐mm Tailor Ring was implanted. Intraoperative water testing confirmed the absence of residual regurgitation (Figures 4 and 5). Although postoperative rehabilitation required time, the overall recovery was uneventful, and the patient was approved for ambulatory discharge on postoperative Day 50. At present, 2.5 years after surgery, there has been no recurrence of MR, and the patient continues regular outpatient follow‐up at our institution every month.

## 3. Discussion

In Japan, TEER with a Class IIa recommendation is indicated for patients with functional MR who present with heart failure symptoms due to impaired left ventricular function despite guideline‐directed medical therapy. For degenerative MR, the procedure is considered with a Class IIb recommendation. Ideal anatomical criteria for TEER include central pathology, no leaflet calcification, a mitral valve orifice area ≥ 4 cm^2^, a mean transvalvular pressure gradient < 4 mmHg, a grasping zone length > 10 mm, normal leaflet thickness and mobility, coaptation depth < 11 mm, flail width < 15 mm, and flail gap < 10 mm. The procedure is not suitable in cases of severe leaflet calcification, extremely short posterior leaflets, active infective endocarditis, or coexistent rheumatic mitral stenosis, since the leaflet becomes extremely difficult to grasp in these situations [1].

Both in western countries and Japan, the success rate of elective TEER is reported to be nearly 100%, and approximately 90% in emergent cases. However, the median time to TEER failure is reportedly less than 6 months, with a mitral valve reintervention rate of 20% and a surgical intervention rate within 1 year of 2%–6%. Among these cases, the incidence of SLDA is reported to be 0.2%–5.1% [2]. Contributing factors to SLDA include short, fragile, or calcified leaflets, as well as difficulty in visualizing the planned clip site with transesophageal echocardiography (TEE), although in some cases the cause remains unidentified [3]. TEE visualization is especially challenging in commissural clipping, and a higher incidence of conversion to open surgery has been reported.

In the present case, the aorta‐hugging trajectory of the catheter resulted in a nonperpendicular orientation relative to the mitral valve and limited posterior reach, leading to less secure grasping of the anterior mitral leaflet (AML) compared with the PML. In addition, clipping performed while the MitraClip NT was entangled with the AML chordae likely resulted in delayed SLDA involving the PML and extensive chordal rupture of the AML. Other potential complications include leaflet injury during clip detachment from the AML as well as enlargement of the left atrium and left ventricle secondary to increased MR severity resulting from the aforementioned mechanisms. These findings suggest that transseptal puncture at the mid‐superior and posterior interatrial septum is particularly important when the target leaflet is located laterally or in patients with limited surgical tolerance.

When SLDA occurs, either repeat clipping or conversion to open surgery becomes necessary. For repeat clipping, techniques involving the placement of a clip on one or both sides of the detached clip have been reported. In our case, there was no leaflet tear or perforation, so repeat clipping could have been considered. However, based on the initial intraoperative challenges, it was determined that placing additional clips on either side of the detached one would be technically difficult, and thus open surgical intervention was selected [4]. The 30‐day mortality rate following open revision after TEER is extremely high, ranging from 10% to 40%, with a 1‐year survival rate of 60%. The rate of successful mitral valve repair is less than 10%. To the best of our knowledge, although numerous cases requiring MVR due to SLDA have been reported [5, 6], we found no previous reports of MVP being performed for SLDA, such as in the present case.

In patients with limited surgical tolerance, shorter operative times are advantageous. In cases where MVP is expected to be time‐consuming or complex, MVR should be considered. From this perspective, MVR has been selected over MVP in open surgery for SLDA because of its greater certainty in controlling MR. However, in general, MVP is associated with better preservation of left ventricular function and improved long‐term survival compared with MVR. In addition, as in the present case, MVP may be a valid treatment option when SLDA is located at the PML and the AML can be preserved, when chordal rupture is repairable using artificial chordae, and in patients in whom the risk of prosthetic valve infection should be avoided, such as those receiving long‐term steroid therapy.

## 4. Conclusion

Patients selected for TEER typically have limited surgical tolerance. Thus, minimizing operative and cardiopulmonary bypass times is essential during open‐heart surgery. However, depending on the etiology of MR and the location of the clip, MVP can also serve as a viable treatment option.

## Author Contributions

Takuro Makiura: writing—original draft. Masahiro Daimon: writing—review and editing. Takahiro Katsumata: conceptualization.

## Funding

No funding was received for this manuscript.

## Consent

Written informed consent was obtained from the patient.

## Conflicts of Interest

The authors declare no conflicts of interest.

## Supporting information


**Supporting Information** Additional supporting information can be found online in the Supporting Information section. Supporting video includes preoperative and postoperative echocardiographic findings as well as intraoperative footage. The video demonstrates SLDA of the MitraClip NT to the P1 segment, en bloc excision of the clip with the involved leaflet tissue, implantation of artificial chordae, commissuroplasty of the anterior commissure, and annuloplasty.

## Data Availability

The data underlying this article are available in the article and in its Supporting Information (Available here).
